# Water Exchange on a Geological Timescale - Examples from Two Coastal Sites in the Baltic Sea

**DOI:** 10.1007/s13280-013-0396-4

**Published:** 2013-04-26

**Authors:** Christin Eriksson, Anders Engqvist

**Affiliations:** 1 DHI Sverige AB, Drakegatan 6, 412 50 Göteborg, Sweden; 2 Aythya AB, Kullasundsvägen 1, 185 37 Vaxholm, Sweden

**Keywords:** 3D-model, Hydraulically coupled basins, Numeric model, Average age of water, Öregrundsgrepen, Kalmarsund, The Baltic

## Abstract

The water turnover of two coastal areas, Forsmark and Laxemar-Simpevarp, has been modeled for 13 selected years between 6500 bc and 9000 ad by utilizing information about past, present, and future bathymetry. The Forsmark area can be described as an open-ended funnel, and is analyzed with a 3D-model (MIKE 3-FM); the Laxemar area is partitioned into clusters of sub-basins treated with a discrete coupled basin model (CouBa). In all simulations, the main variation factor is the land uplift. The 3D-model domain is successively modified. For the CouBa approach the successive basin configurations are objectively deduced based on the 3D domain modifications. The average age (AvA) of the resident water relative to the open coast is generally lower for the Forsmark area. A typical progression is that the AvA values increase until a sub-basin ceases to be connected to the coastal zone. This disconnection is often preceded by a lowered AvA.

**Electronic supplementary material:**

The online version of this article (doi:10.1007/s13280-013-0396-4) contains supplementary material, which is available to authorized users.

## Introduction

In almost all endeavors dealing with coastal waters the turnover time becomes a central aspect (e.g., Kremer and Nixon 1978; Csanady 1982; Stigebrandt 2012). Most often, such studies are performed with a bathymetrically fixed coastal section being constant in time while being subjected to varying forcing. In the present study, however, the bathymetry varies during an extended time period while the forcing, for lack of historical and future data, mainly remains constant. The objective is to estimate the passage time through near-shore coastal waters of radionuclides that could potentially be released from hypothetical repositories in the geosphere, and that via terrestrial ecosystems—implicitly including streams, lakes, and direct runoff—or directly enter waters connected to the coastal zone. After leaving this zone, the radionuclides are for obvious reasons considered to be rapidly diluted in the greater water body of the Baltic Sea. The focus is thus on their radiological impact on humans making use of resources from the coastal zone. It must be emphasized that here the water turnover is in focus. The additional complication of radionuclides attached to particles subjected to sedimentation processes is treated by Corell and Döös (2013).

Originally there were two competing sites for the nuclear repository, the Forsmark and Laxemar-Simpevarp areas. Although the former has now been selected, it has been a policy that both candidate sites should be equally thoroughly investigated. Since they differ considerably from a coastal oceanographic point of view, two different models have been employed. This article is a synthesis compiled by merging material from two separate technical reports (Karlsson et al. 2010; Engqvist 2010), which provide greater technical detail.

A schematic diagram of the data dependencies is depicted in Fig. S1 (in Electronic Supplementary Material).

## Materials and Methods

### Water Turnover Accounting

There are several different methods to account for the rate of water exchange. Jönsson et al. (2004) compared the ‘mass balance budget’ method with the trajectory-based ditto for two large Baltic coastal bays. Döös and Engqvist (2007) evaluated the trajectory method and a tracer-based approach comprising the age of water parcels inside a defined coastal section along the contemporary Laxemar-Simpevarp coast. The age was measured relative to the exogenous coastal water. They found that the two methods gave mainly the same results. The age-based method has the advantage of being an easily implemented code into the numerical models. The results of the two studies are thus expressed as specific average age (AvA) of water, i.e., age per volume, with the age measured relative to the exogenous water in the coastal zone; see Engqvist et al. (2006) for an account of this measure of residence time and its relationship to other such measures. The mathematical definition (Engqvist 1996) is

1 $$ \frac{{{\text{d}}a}}{{{\text{d}}t}} = K\frac{{\partial^{2} a}}{{\partial t^{2} }} + 1, $$

where *a* denotes the AvA scalar. The right-hand side is the substantial time derivative that includes advection, *K* is the diffusivity parameter and the aging, one time unit per unit residence time, is produced by the trailing unity source term on the right-hand side. The boundary condition is that AvA values of exogenous water are set to zero.

The AvA values of the individual biosphere objects (BOs) that are finally presented thus mainly represent the age of the water relative to the open coastal zone. For the Laxemar-Simpevarp area, a conservative but realistic estimate of the AvA of the coastal zone may subsequently be added to the AvA of all entailed BOs. For the Forsmark area, the direct and wide contact with the open sea along the northern boundary is deemed to make such correction unnecessary. These AvA data thus represent a refined abstraction of coastal oceanographic dynamics as yearly averages spanning an extended period of time and are the results that are passed on to the landscape modeling of radionuclide transport (Avila et al. 2013)

### Employed Models

Three numerical models were employed in this study. (i) The major 3D-model computation effort related to the Forsmark area for the last 2000 years and involved MIKE 3FM (DHI 2011a, b; Butts and Graham 2008). An overview presentation of its features is given in Box S1 (in Electronic Supplementary Material). (ii) Another 3D-model, AS3D, was used for the same area when it was completely submerged during earlier (bc) times (Box S2 in Electronic Supplementary Material). (iii) The third model is a hydraulically coupled discrete basin model, CouBa, which was designed for resolving the water exchange in morphometrically complex water areas in particular archipelagos, by assuming horizontally well-mixed conditions in the basins and by focusing on the water exchange through the interconnecting straits (Engqvist and Stenström 2003, 2009), see Box S3 (Electronic Supplementary Material).

### Study Areas

**Fig. 1:**
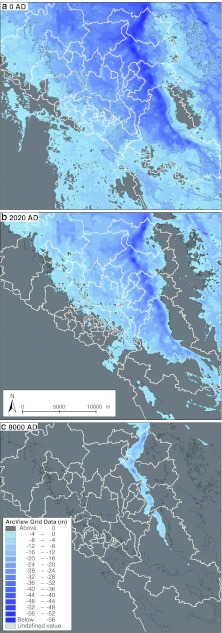
The partitioning of the Forsmark area into BOs exemplified by three time periods: **a** 0 AD, **b** 2020 AD, **c** 8000 AD. For 3000 bc all BOs fell in the open coast and the corresponding AvA values were computed separately, see Box S2 (Electronic Supplementary Material)

**Fig. 2:**
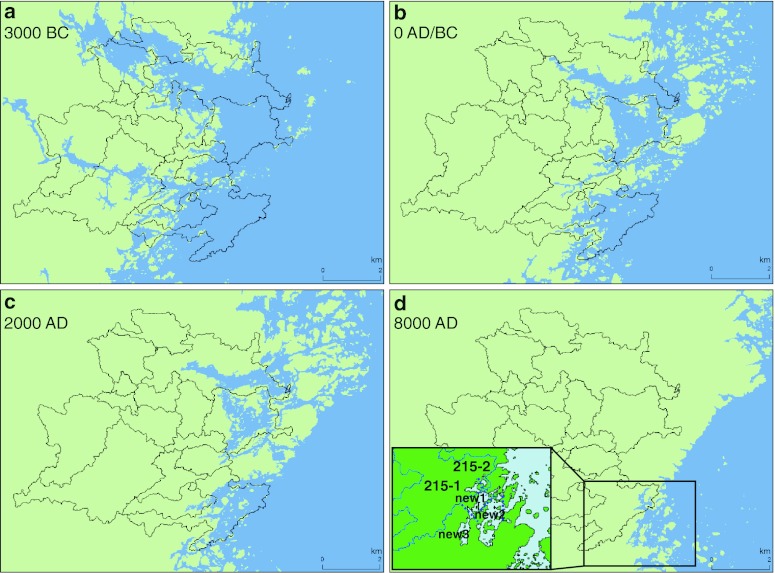
The analogous subdivision into BOs as in Fig. 1 of the Laxemar-Simpevarp area for the same time periods plus 3000 bc. As the land-lift progresses, the last remaining BOs connected to the open coast are found to the south. For 8000 ad the newly occurring basins connecting to the coastal zone (not being BOs) are designated ‘new1’, ‘new2’, etc.

The contemporary Forsmark water area (Öregrundsgrepen) is like an open-ended funnel (Fig. 1b) with its wider and deeper opening facing north, whereas the concomitant coast at Laxemar-Simpevarp (Fig. 2c) in the northern Kalmarsund is more rugged and from a morphometrical point of view more complex, forming a number of more or less land-locked embayments. Both areas are subject to comparably feeble stratification, windforcing enhanced by extended fetches in some directions, and seasonal heating/cooling with deepening summer thermocline and possible ice formation during the winter period (Stigebrandt 2001).

Both present study areas have been partitioned into topographically defined ‘biosphere objects’ (BO), i.e., the areas most likely to be affected by a potential release of radionuclides (cf. Lindborg et al. 2013). Such BOs have in common inferred exit points of potentially discharged radionuclides that may reach the surface land and water ecosystems in the vicinity of the planned (Forsmark) or hypothetical (Laxemar-Simpevarp) locations for a nuclear geosphere repository. The delimitation of the BOs for Forsmark (Kautsky et al. 2013, their Fig. 3) is strictly based on catchment-area features and this applies also for Laxemar (Fig. S2a in Electronic Supplementary Material). The Forsmark BOs are enumerated 100 through 151; the Laxemar-Simpevarp ones 200 through 217, but the last small BO was subsequently conjoined with BO 216.

The exact locations of the inferred exit points (Fig. S2b in Electronic Supplementary Material) matter only at Laxemar-Simpevarp where they give the possibility of excluding the needless calculation of results for some BOs with no direct connection with these points. Only the locations for which the geosphere pathways enter directly into basins connected to the coastal waters (Berglund et al. 2013) need to be computed. For the Forsmark area, all basins connected to the coastal zone are tacitly included regardless of the exit points.

All basins go through a succession of being a part of the open coastal zone, becoming gradually more landlocked, to eventually become a part of the land ecosystems. In this progression, some BOs become lakes detached from exchange with the coastal zone (Table S1, Electronic Supplementary Material). For the time period over which they are connected to the coastal zone, they are subjected to sedimentation processes which together with the land-lift dynamics have been computed by Brydsten and Strömgren (2004), Brydsten (2006) and Lindborg et al. (2013). Some instances of the shoreline are depicted in Figs. 1 and 2 and the Digital Elevation Models (DEMs) are used for computing the bathymetries for each of the modeled year.

### Bathymetric Data

The bathymetries for the different time periods were provided by Umeå University and needed to be preprocessed for use in the models. For the Forsmark area this involved the same kind of triangulation as is shown in Box S1 (Electronic Supplementary Material). For Laxemar-Simpevarp, each new *sub*-*basin* (SB) that arose had to be identified together with its interconnecting straits and the hypsographies of all these emerging landscape elements needed to be evaluated from the given bathymetrical GIS data. The specified procedures are listed in Box S4 (Electronic Supplementary Material) concerning the basins and in Box S5 (Electronic Supplementary Material) for the straits. A few examples of the resulting basin and strait configurations are shown in Figure S3 (Electronic Supplementary Material).

### Forcing

Both coastal sections are subjected to forcing that alters the sea level by internal discharge of freshwater and by an externally fluctuating sea level. If the water were of homogeneous density, the induced sea level differences would suffice for a complete computation of the water movements employing a quadratic resistance law for the strait connections (barotropic forcing).

Sea water is, however, rarely densimetrically homogeneous, but is stratified with normally less dense water on top of a denser layer. In estuaries where fresh water is discharged into brackish water, estuarine circulation arises. The lowered density inside the estuary then facilitates a return current bringing in denser bottom water by a current going in the opposite direction of the out-flowing surface current. The stratification of external water outside the coast varies in time. On upwelling occasions, denser water is known to force its way as a bottom current displacing the surface water that was forced to exit. The two latter density-dependent processes are called baroclinic forcing and most often they dominate the water exchange. An exposé of water exchange processes has been compiled by Stigebrandt (2012) summarizing about 40 years of his research.

The models needed to be driven by a forcing that represents the seasonal change during a calendar year. Since sufficiently detailed historical and future forcing information cannot be obtained, the year 2004 was used as forcing for all model runs except for the Forsmark area where a different forcing has been applied for the years previous to 0 ad. In Figure S4 (in Electronic Supplementary Material), an overview of the forcing factors is shown. With the extended wind fetches in certain directions applicable to both areas, the tilting of the water surface by the wind drag (set-up) may be considerable. Also, on coastal scale the Ekman dynamics contributes as does the induced wind-mixing on the local scale. In Figure S4a it is clearly seen that as a yearly average, the predominant wind direction is from the southwest for both areas. To determine the heat exchange with the atmosphere in the local model, air temperature, humidity, and cloudiness are required input data. The observed time series for 2004 from the station ‘Örskär A’ was used in the Forsmark model, whereas for Laxemar-Simpevarp the station ‘Ölands Norra Udde’ was used.

The resulting windforcing on other scales than local manifests itself in the sea-level forcing (Fig. S4b, Electronic Supplementary Material). For both model areas, this was provided by running the AS3D model for the entire Baltic (Engqvist and Andrejev 1999, 2008; Engqvist 2006). These diagrams are largely similar, reflecting the sea level of the entire Baltic as forced by the model at the Kattegat boundary. For Forsmark a comparison with measured sea level near land is shown.

From studies of other Nordic coastal embayments, the baroclinic exchanges in upwelling events are the most efficient water exchange processes (Stigebrandt 1990; Engqvist and Omstedt 1992). The boundary forcing of the local models thus also involves salinity and temperature profiles, which together determine the density (Figure S4c, d, Electronic Supplementary Material) at one local station for Laxemar-Simpemarp and for the Forsmark southern and northern boundaries.

Two streams discharge into the inner part of Forsmark area (Kallrigafjärden) with a combined freshwater flux of circa 10 m^3^ s^−1^ as a yearly mean (Fig. S4e, Electronic Supplementary Material). The flow varies considerably with a marked peak in the springtime. A notable estuarine circulation mode is thus present in this directly receiving embayment most of the time, but is barely detectable in greater Öregundsgrepen. These levels of discharge have been maintained since the main catchment inland area is generally unaffected by the land uplift. The contemporary stream run-off in the Laxemar-Simpevarp area is about one order of magnitude smaller in comparison. The catchment areas vary considerably over the modeled time-frame, so for each year their areas have been separately assessed and combined with the run-off rate of present time, which rate has been assumed constant between the modeled years.

## Results

After an appropriate spin-up time (1-year cycle for the Laxemar-Simpevarp area, and 1 month for the Forsmark area) each of the two models was run one full year for each of the selected times.

### Forsmark Area

For the Forsmark area, the vertically integrated AvA of the entire Öregrundsgrepen is shown for three ad time periods in Figure S5 (Electronic Supplementary Material). Overall, the AvA increases with time as the area becomes shallower and more isolated from the Baltic Sea. However, there are variations along the way due to different factors. By analyzing the AvA for the different time steps, the evolution of the Öregrundsgrepen can be divided into three different stages that have different types of main water exchange. The first stage is between 6500 bc and 0 ad, when the Öregrundsgrepen area is located in the open sea without any physical boundaries restricting water exchange (Box S2, Electronic Supplementary Material). There is no significant difference in the water exchange, as indicated by the AvA values, between these three bc 1-year periods. The value for the entire Öregrundsgrepen is between 5 and 7 days, and the basin values are of the same order of magnitude, with extremes of 2 and 10 days. This is the phase during which the smallest AvAs in the evolution of Öregrundsgrepen are found.

The second stage is between the years 0 ad and 3000 ad, when the southern boundary of Öregrundsgrepen has narrowed due to land uplift thereby restricting the water exchange. The narrowing of the southern boundary results in higher AvA values compared with the earlier stage. The net flow through the southern boundary into the Baltic in the 0 ad simulation is reduced by a factor of four. Similarly the net flow into Öregrundsgrepen through the northern boundary decreases by almost the same factor. These combined effects should increase the residence time for the basin but the AvA later decreases as a result of a complex interplay between a narrower southern boundary, decreasing basin volumes and the cross-sectional areas between adjacent basins.

The AvA varies between 8 and 34 days, with the mean for the entire area increasing from 13 up to 25 and then decreasing to 14 days again. In general, the shallow western basins have higher AvA values than the deeper eastern basins. During the third stage, basins are gradually becoming more enclosed and are one by one transformed into lakes. The AvA for the entire area increases from 14 to 43 days, but with a wide spread between basins, varying from 13 to 105 days, where the innermost basins in the system have the highest AvA values.

However, between the years 1000 ad and 3000 ad the AvA decreases, probably due to smaller basin volumes combined with inflows of the same magnitude as for the year 1000 ad. The third and last stage of the Öregrundsgrepen begins in the year 4000 ad when the land has raised enough to close the southern boundary. From that point on the AvA starts to increase again, since exchange can occur only over the northern boundary. As a consequence of the change into a one-ended system, the inner parts of Öregrundsgrepen only experience very restricted water exchange.

When analyzing the AvA of individual basins in terms of their distance to the open sea, there is a strong co-variation. Also, the mean depths of basins have been compared against AvA and show that shallow basins have higher AvA values, which is not surprising as the largest flows occur in the deeper basins situated along the eastern boundary. Where the small rivers Olandsån and Forsmarksån discharge into Öregrundsgrepen, the AvA value is somewhat smaller than in surrounding basins. This effect of locally decreased AvA, due to freshwater discharge, increases with time as the entire Öregrundsgrepen shallows.

### Laxemar-Simpevarp Area

For the Laxemar-Simpevarp area, the volume averages of the AVAs for the SBs are presented in Figure S6 (Electronic Supplementary Material) for four samples of the 13 time periods. These values must subsequently be recalculated by computing their conjoined AvA volume average so that they refer to the AvA values of the corresponding BOs, which are the relevant accounting entities to be passed on to the landscape dose computation (Berglund et al. 2013). It can be seen that for some SBs (e.g., 205 for 3000 bc) the AvA value does not attain a steady state within the modeled year. With iterated year-long runs, it would eventually reach a quasi-equilibrium, but with such large AvA values the water area in question should be considered as a lake with a one-way connection to the coastal zone.

**Fig. 3:**
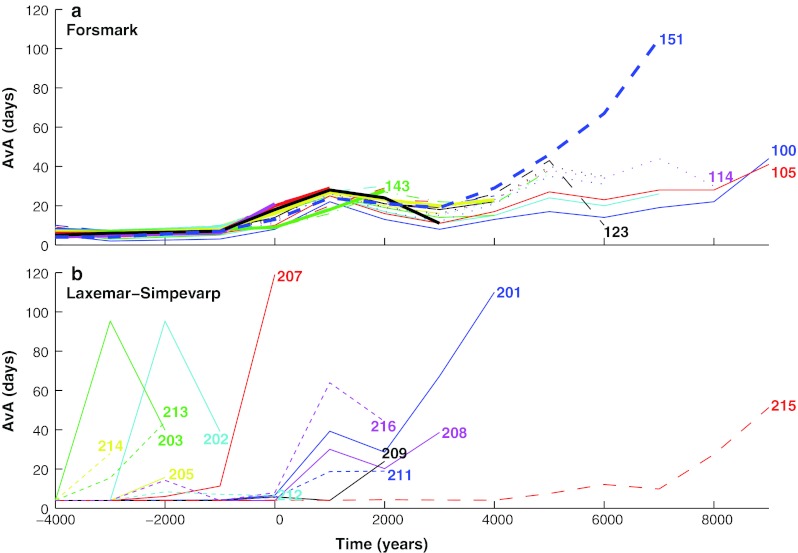
Overview of the resulting AvA values as a function of time. **a** For the Forsmark area with a few BO numbers indicated by the end of the time period when the corresponding SB ceases to be in exchange contact with the coastal zone. **b** For the Laxemar-Simpevarp area, the BO number is analogously indicated. The enhanced degree of land-lock of this area results in generally higher AvA values

A conservatively estimated correction of 4 days for the AvA of the coastal zone was added (Engqvist 2010) and the SBs of the numerical model results were recalculated to volume-average equivalents pertaining to the respective BOs. This permitted the results for the two areas to be compared (Fig. 3a, b). It can be seen that the AvA values of the Laxemar-Simpevarp area are generally greater than for the Forsmark area even though there are also for many time periods BOs with comparatively low AvA values, reflecting an intense water exchange with the coastal zone by straits with large cross-sectional areas.

For the Laxemar-Simpevarp area BOs 204, 205, 207, 211, 212, 213, and 215 the AvA values increase monotonously before the two-way exchange with the coastal zone ceases to exist successively at different time periods. This is contrary to the BOs 202, 203, 208, and 216 which display a maximum while they are still coastal basins with two-way exchange with the coastal zone. BO 201 is an intermediate between these two categories with a peak that is followed by a renewed AvA increase. The remaining BOs are not associated with discharge locations or they exist for only one time period.

## Discussion

That the most complex basin configuration for both areas occurs for contemporary times may seem somewhat surprising. A plausible explanation is that the nuclear reactors must be located near the present-time shoreline to arrange for the cooling. This in turn has decided the location of the repository and influenced the estimated radionuclide discharge areas. If the placement had been performed in the vicinity of a past or future shoreline, this would have meant a reduced present complexity due to the ongoing transformation (Table S1, Electronic Supplementary Material) from open coast to land via the intermittently occurring coastal basin. As the basin configuration is primarily determined by the bathymetry, it could also be argued that the shallow bathymetry is better resolved, which would give smaller-scale distinctions in basin characteristics.

### Validation

Concerning the Forsmark area, the model results for the present day are in good agreement with observed sea levels and temperatures. The agreement is not good when comparing modeled and observed salinities, but the discrepancy is mainly an almost constant offset. The model captures most of the variability in the observations. Since it is the differences in the density stratification—and not the absolute values—that induce water movement, the model is expected to produce realistic estimates of the water exchange. It should be mentioned that there are questions about the quality of both measurements and the forcing data (Engqvist and Andrejev 2008) from the Baltic Sea model. Hence, it is difficult to quantitatively determine the quality of the local model, particularly as it is very much dependent on the realism of the forcing. A comparison of the present study with an earlier study (Wijnbladh et al. 2008) in which the AS3D model was forced with data from 1988 convincingly showed a striking consistency of the yearly mean volume flows in and out of the Forsmark BOs.

### Uncertainty Due to Hypsography

In spite of the pronounced ambition to treat all derivations of the hypsographic data as similarly and objectively as possible (Boxes S4, S5, Electronic Supplementary Material), there are many possible sources of errors in addition to the finite resolution of the DEM, which can only be compared with an almost independent data set for 2000 ad. Some of the SBs in a former study (Engqvist 2006) were, in addition to being derived from a DEM, also based on in situ soundings. In particular this applies to the narrow straits that both the present and the former DEMs resolve poorly. Some of the basins of these two studies are sufficiently close counterparts, so a direct comparison of their AvA values offers a way to assess the overall inaccuracy with regard to the hypsographic aspect. This was achieved by running the present version of the CouBa model with the configuration and hypsographic features of the former study (Engqvist 2006) and after adjusting the AvA volume averages of the former SBs so that they correspond to as a close match as possible to the present ones.

The result is presented in Figure S7 (Electronic Supplementary Material) as a scatter plot of the corresponding AvA values when comparing the six most geographically coincidental SBs. The correlation coefficient is 0.95. The slope of the regression line for the average values is about 40 % less than unity, meaning that in the present study the mean AvA values are comparatively underestimated relative to what was found in the former study. Unfortunately this is not consistent with the project guideline to avoid overestimation of the rate of water exchange, since this translates into possible underestimations in the ensuing dose computations. Since it cannot be determined which of these two studies is the more realistic one, it must be concluded that there is an uncertainty of the same degree concerning the overall methodology in deriving the bathymetric data.

### Sensitivity Analysis

For both areas, a sensitivity analysis based on the included forcing factors has been performed in order to evaluate their relative importance for the determination of AvA values. For the Forsmark area, this sensitivity analysis has been made for the years 2020 ad and 5000 ad. It was carried out by removing completely one of the major forcing mechanisms at a time and comparing the results for the AvA with the reference simulation. The results show that the present-day stage, when Öregrundsgrepen is open-ended, is primarily forced by the wind and sea levels. After the southern entrance is closed and Öregrundsgrepen has been transformed into a bay, the baroclinic forcing due to variations in stratification and runoff from land comes into play, whereas the barotropic forcing becomes insignificant. Wind still plays an important role, though. As can be expected, the AvA increases as a forcing mechanism is removed, with some local exceptions. Overall the sensitivity of the AvA values to the complete removal of a forcing mechanism was less than 50 % in relative terms or less than about 10 days in absolute terms. Some specific basins show higher sensitivity, at least for parts of the year, such as basin 118 where AvA approximately was doubled when the wind was removed.

Engqvist (2006) performed a sensitivity analysis of the CouBa model pertaining to the Laxemar-Simpevarp area in which not only the hypsographic data (varying basin areas and strait cross-section areas) but also ±10 % perturbations of the forcing factors (run-off, wind speed, boundary and sea-level fluctuations) were studied, in total eight different factors. It was found that reducing basin areas and the run-off intensity meant a reduced AvA of almost the same magnitude, averaged over the total water volume for all 12 SBs. Adding a high-frequency component superimposed on the 2-h Nyquist frequency barotropic forcing gives a noticeable lowering (20 %) of the AvA for the secluded basins in the Laxemar-Simpevarp area, but there are no reasons to assume that such manipulation would affect the more open Forsmark area to the same extent.

### Uncertainty Due to Climatology

Notwithstanding other sources of uncertainty, there is good reason to believe that the interannual climatologically induced variations during the 12 000 years that the present study spans are less than the intraannual seasonal variations during 2000 ad. A first but conservative worst-case appreciation of the uncertainty due to climate variations would be to subtract one standard deviation from the mean AvA values and assess the corresponding slope (Fig. S7, Electronic Supplementary Material). The resulting slope is close to 1/3, making a conservative estimate of the overall uncertainty a factor 3 for the Laxemar-Simpevarp area. It seems safe to infer that this estimated uncertainty factor should be sufficiently conservative to also cover the corresponding uncertainties for the Forsmark area.

## Conclusions

It is found that the more open Forsmark area displays generally a more rapid water exchange than the more secluded and land-locked basins of the Laxemar-Simpevarp area. Common to both areas is that the AvA-measure of many individual sub-basins peaks before the sub-basin ceases to have a two-way flow connection to the coastal area.

## Electronic supplementary material

Below is the link to the electronic supplementary material.

Supplementary material 1 (PDF 2723 kb)
