# Supplementary material for: Water Exchange on a Geological Timescale - Examples from Two Coastal Sites in the Baltic Sea
Source: Ambio. 2013 Apr 26;42(4):447–54. doi: 10.1007/s13280-013-0396-4 (PMC3636375; doi:10.1007/s13280-013-0396-4)
Supplement: Supplementary file 1 — Supplementary material 1 (PDF 2723 kb) [file 13280_2013_396_MOESM1_ESM.pdf]

Electronic Supplementary Material

Water Exchange on a Geological Timescale - Examples from Two Coastal Sites in the Baltic Sea

Christin Eriksson and Anders Engqvist

SUPPLEMENTARY FIGURES AND TABLE

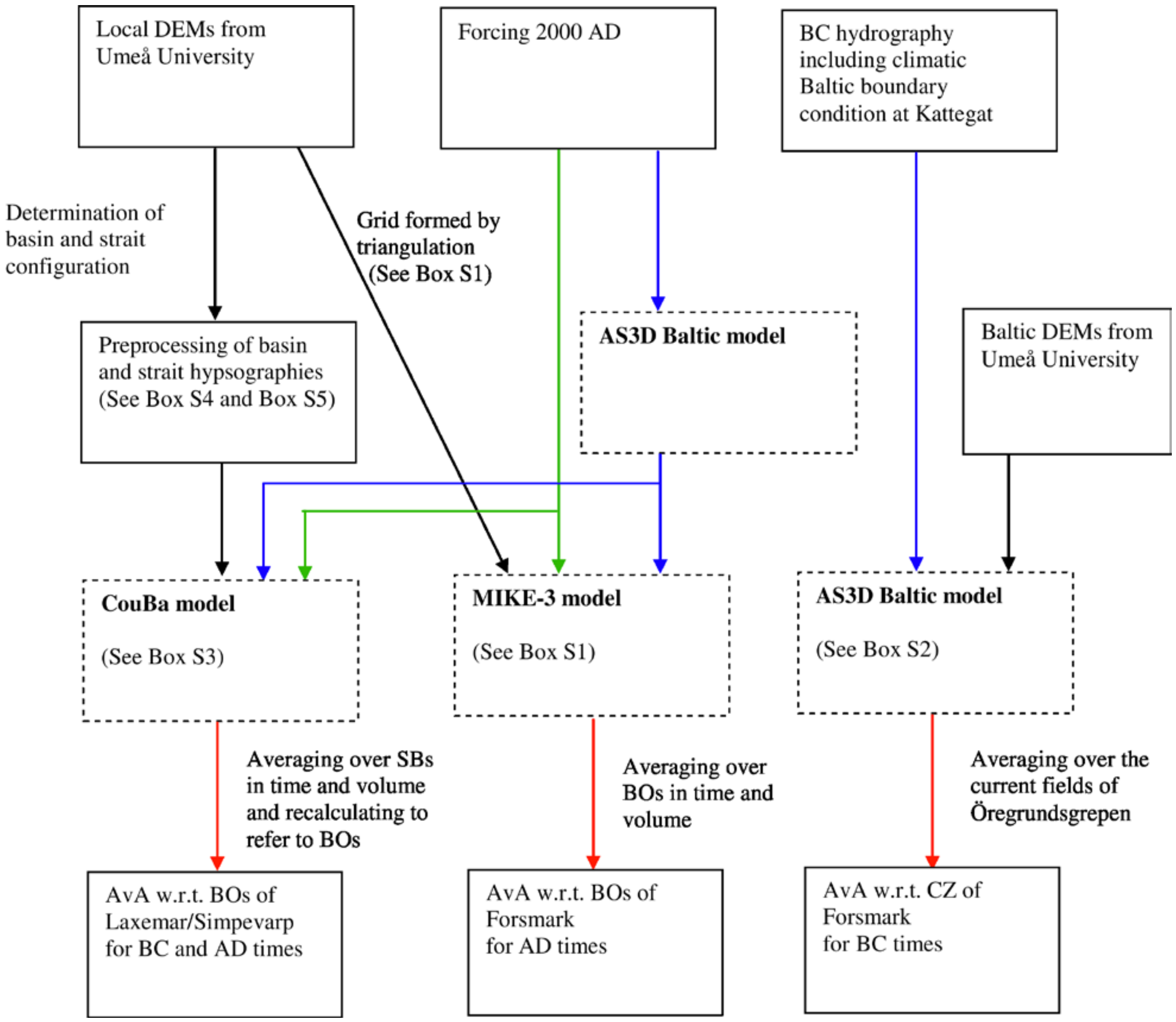

Fig. S1 Schematic block diagram showing the data dependencies of the models and the pre- and postprocessing computations. Black arrows denote bathymetric data, green contemporary (2004 AD) meteorological and hydrological forcing, blue hydrographic forcing (boundary salinity, temperature and sea level data) for BC times, and red arrows the resulting AvA data. CZ stands for Coastal Zone

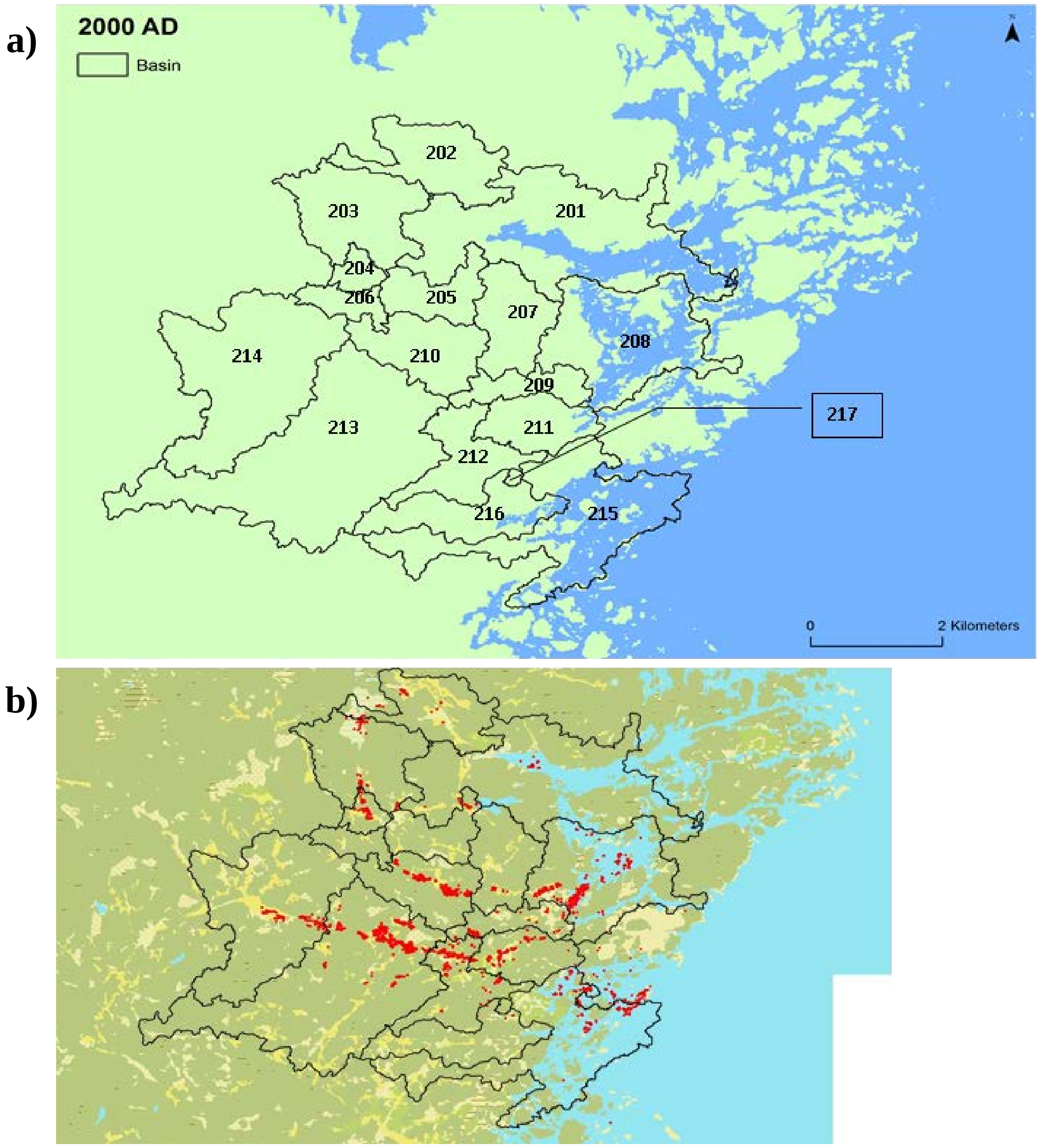

Fig. S2 a) Subdivision into biosphere object (BO) basins enumerated 201 through 216 for the Laxemar-Simpevarp area. The sea level shown is 1m above the present time (2000 AD) yearly mean water level (MW). The minute BO 217 was defined at an early stage of the study but was subsequently conjoined with BO 216. **b)** Location of the anticipated exit points in the Laxemar-Simpevarp area for radionuclides released from a hypothetical repository via the geosphere indicated as red spots

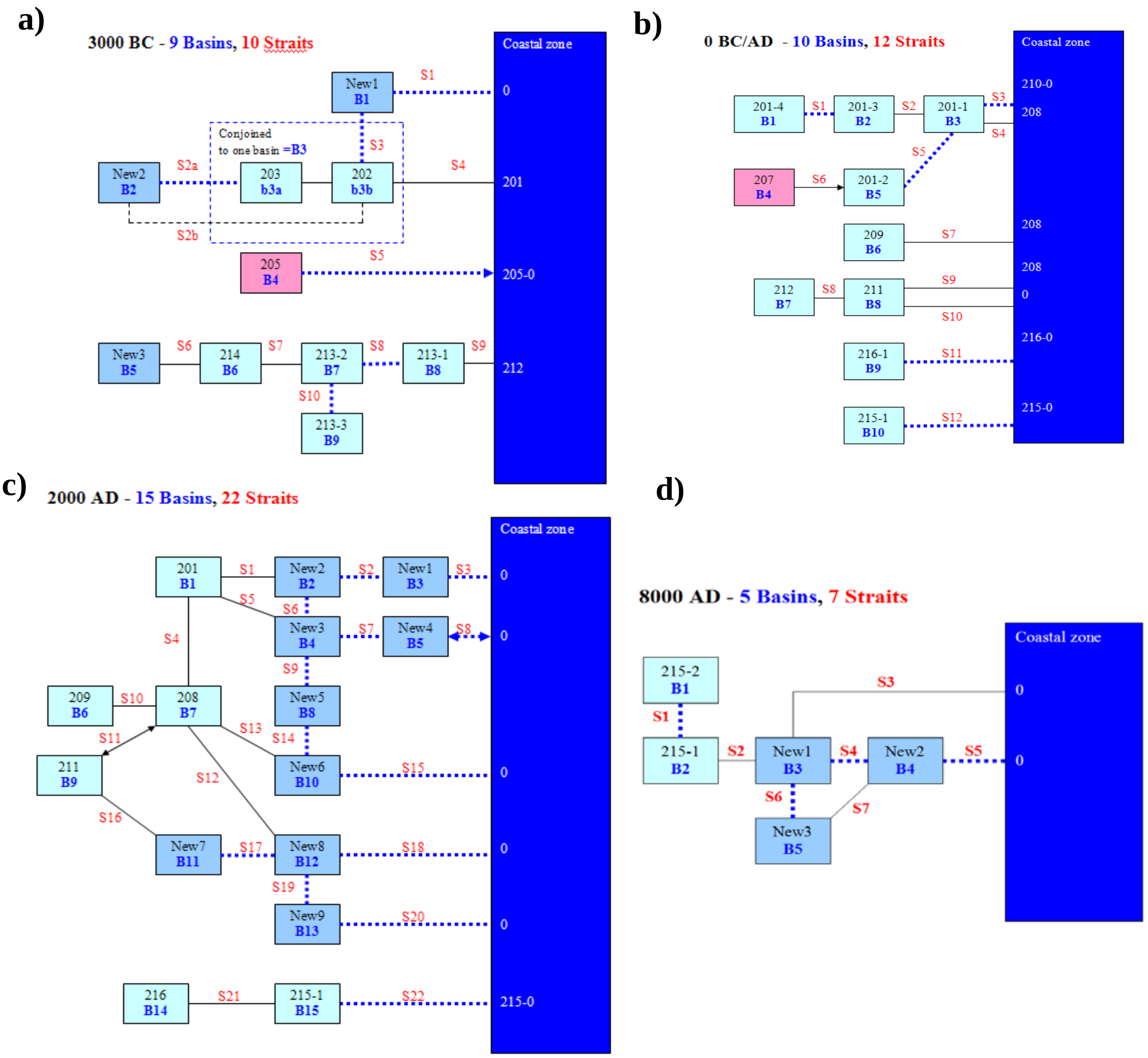

Fig. S3 a) The resulting coupled basin configuration for 3000 BC following the prescribed procedures in Box S4 and Box S5, with the BOs named in black font and the systematic modeled basin names in bold blue fonts. The model naming of straits is given in red labels. Some new basins that are not BOs are included for oceanographic realism. The wide cross-section between BOs 202 and 203 motivates their conjoining. The red-colored basin proved in retrospect to belong to the catchment area due to an elevated sill height that exceeds the highest occurring sea level. **b)** The configuration for 0 BC/AD with the same color code as in Fig. S3 a. **c)** The contemporary time configuration (2000 AD), for which the number of basins and straits peaks. **d)** The configuration at 8000 AD with considerably reduced complexity. For all configurations the thresholds that coincide with the borders of the BOs are drawn with a black line; all other straits are depicted with a broken blue line. A single arrow indicates unidirectional flow for the entire year; a double arrow means the same but at different times during the one-year long simulation. In such a one-way exchange, fluxes are controlled by the actual sill height of the strait

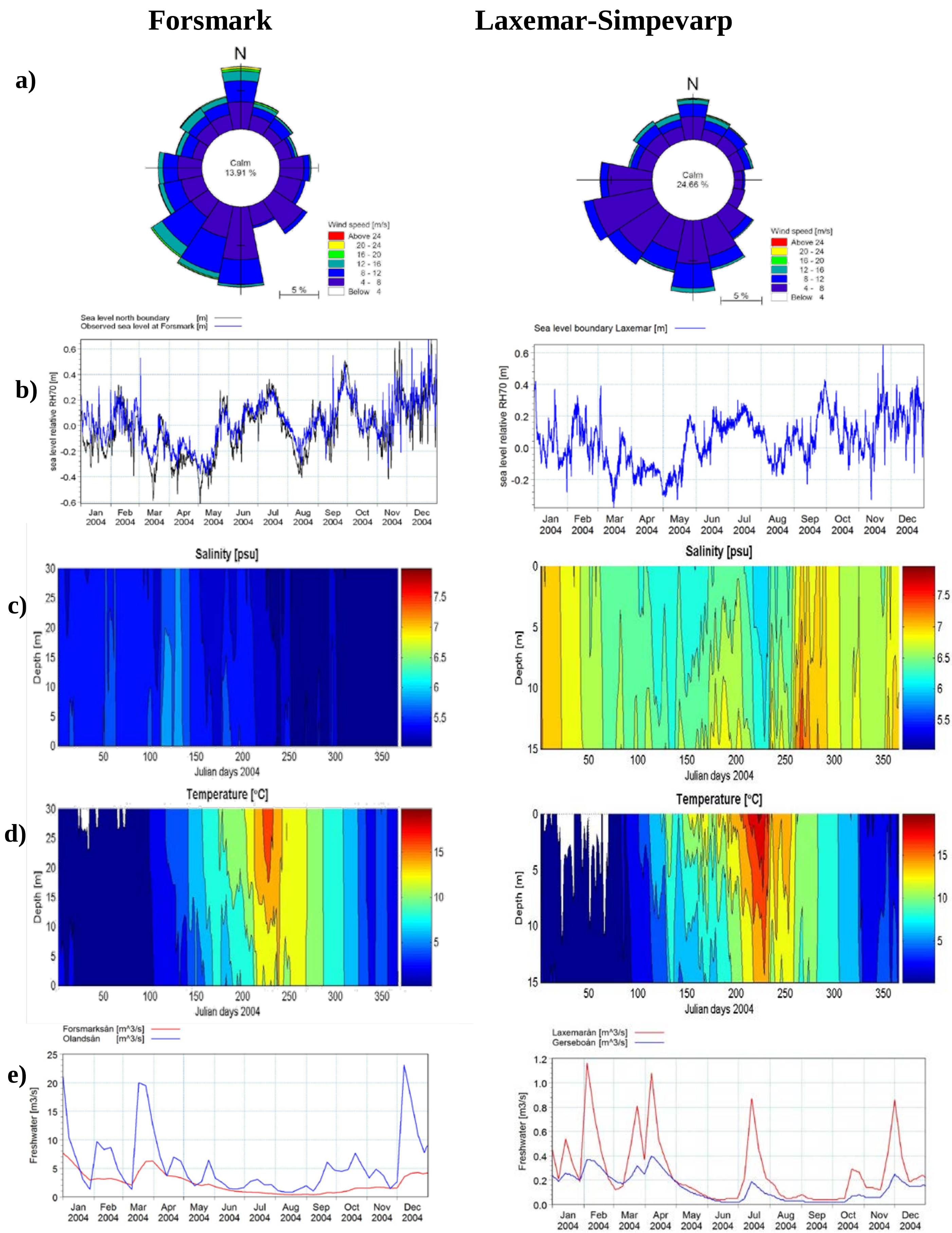

Fig. S4 Overview of the forcing of the two models. All data pertain to 2004. **a)** Wind forcing. **b)** Sea-level fluctuation measurements including comparison to modeled forcing for the Forsmark area. **c)** Boundary salinity contours. Note the considerably lower salinity in the Forsmark area. **d)** Boundary temperature contours. White contours represent water with marginal sub-zero temperature. **e)** Freshwater run-off by streams

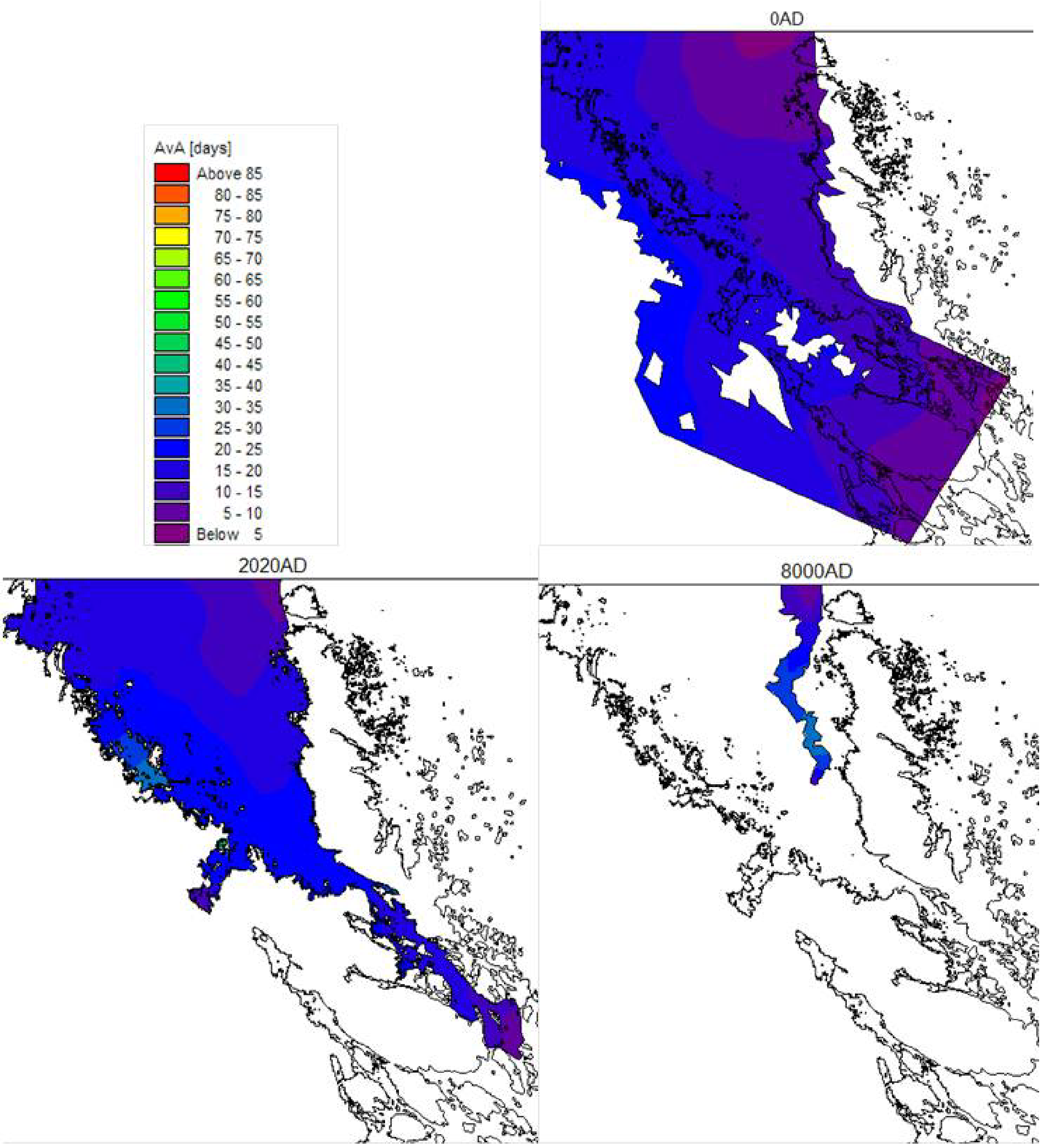

Fig. S5 Horizontal distribution for Forsmark of the vertically volume averaged AvA for the same three instances of the simulated AD years as in Figure 1 of the main (printed) document

a)

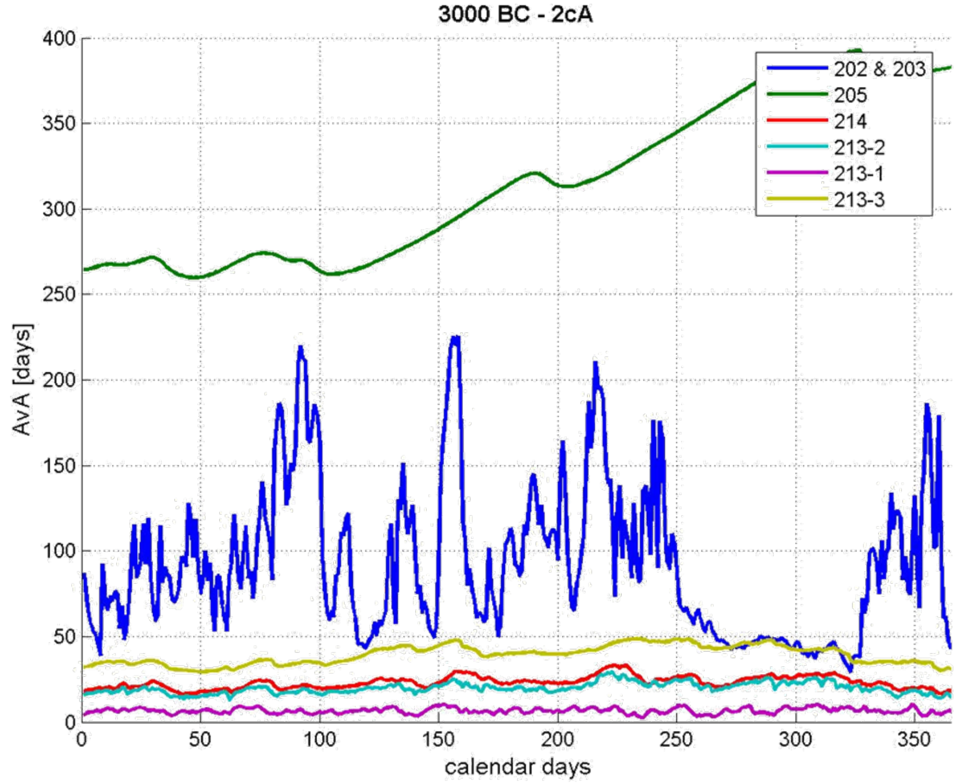

b)

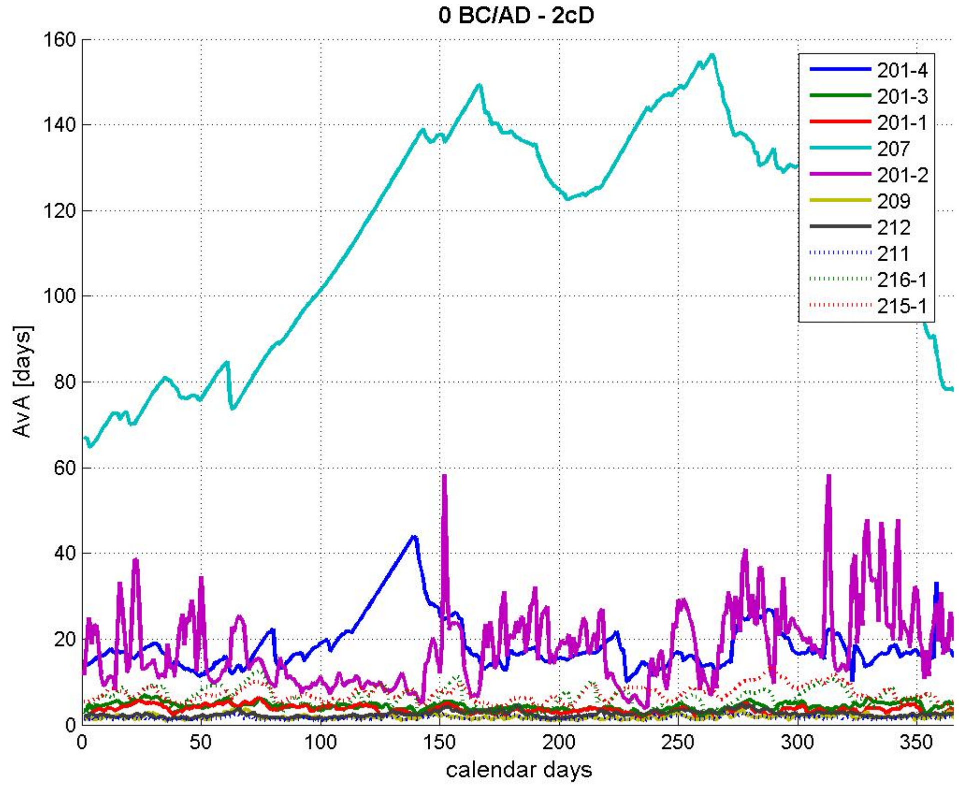

Fig. S6 The volume averaged AvA-variation for Laxemar of the ensuing sub-basins (SBs) exemplified by the same four instances of the 13 one-year long runs as in Figure 2 of the main document. On this page: **a)** 3000 BC; **b)** 0 BC/AD. Next page: **c)** 2000 AD; **d)** 8000 AD. The basins that do not reach a quasi-equilibrium in a year cycle (e.g., BO 205 for 3000 BC) have been discarded since they more rightly should be considered as lakes with no two-way connection to the coastal zone

c)

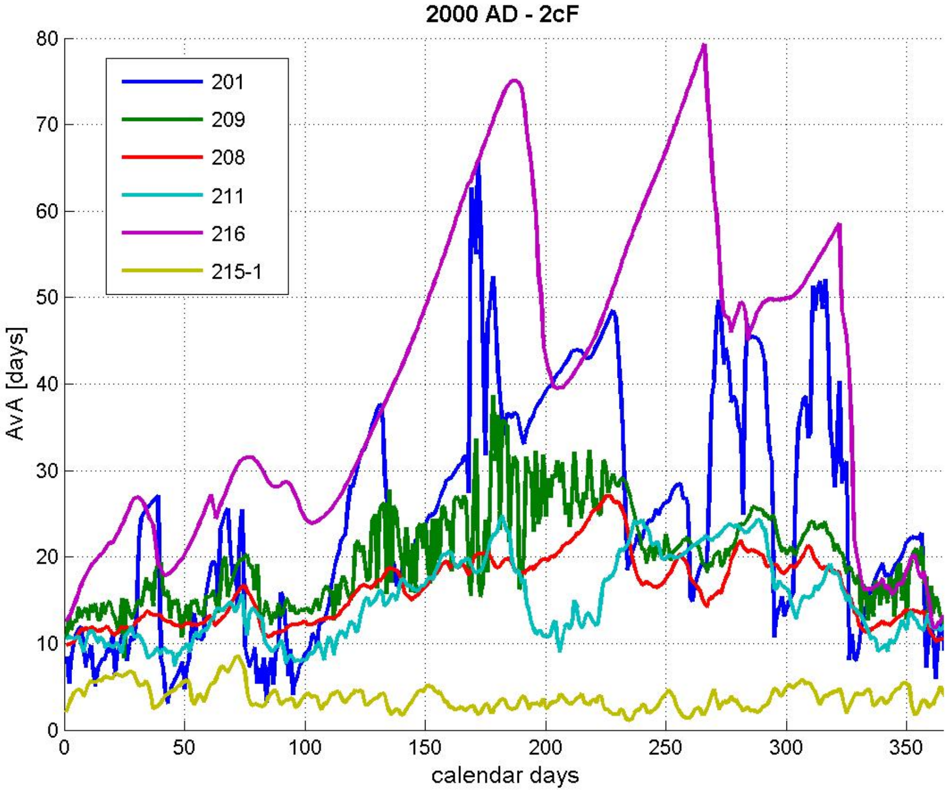

d)

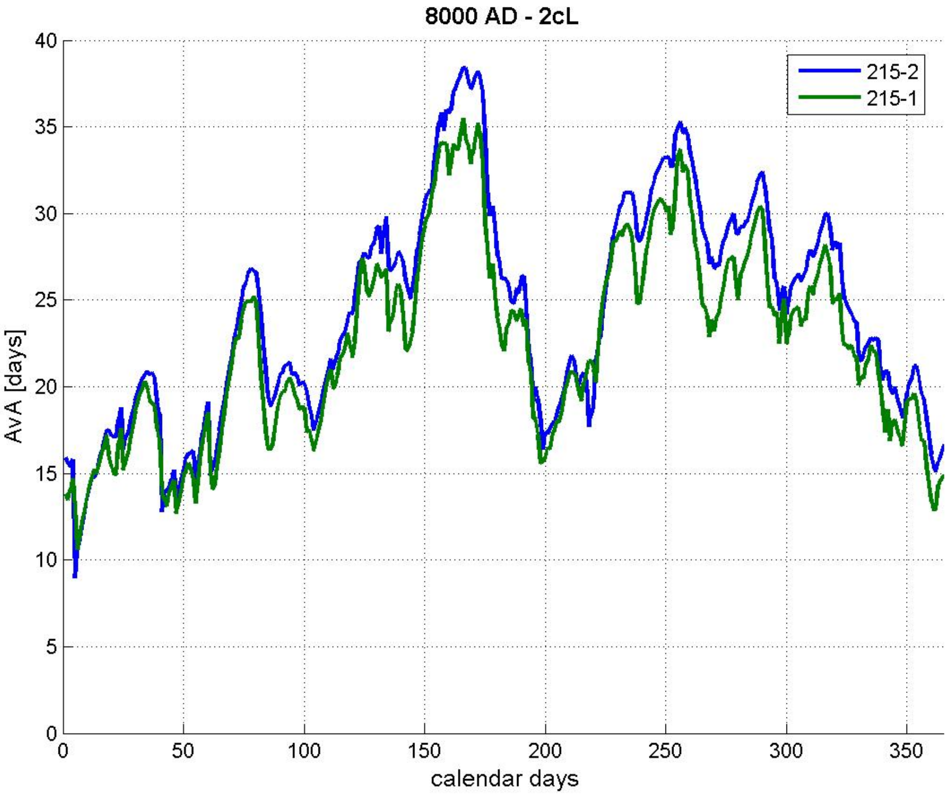

Fig. S6 c, d Complete Figure S6 legend, see previous page

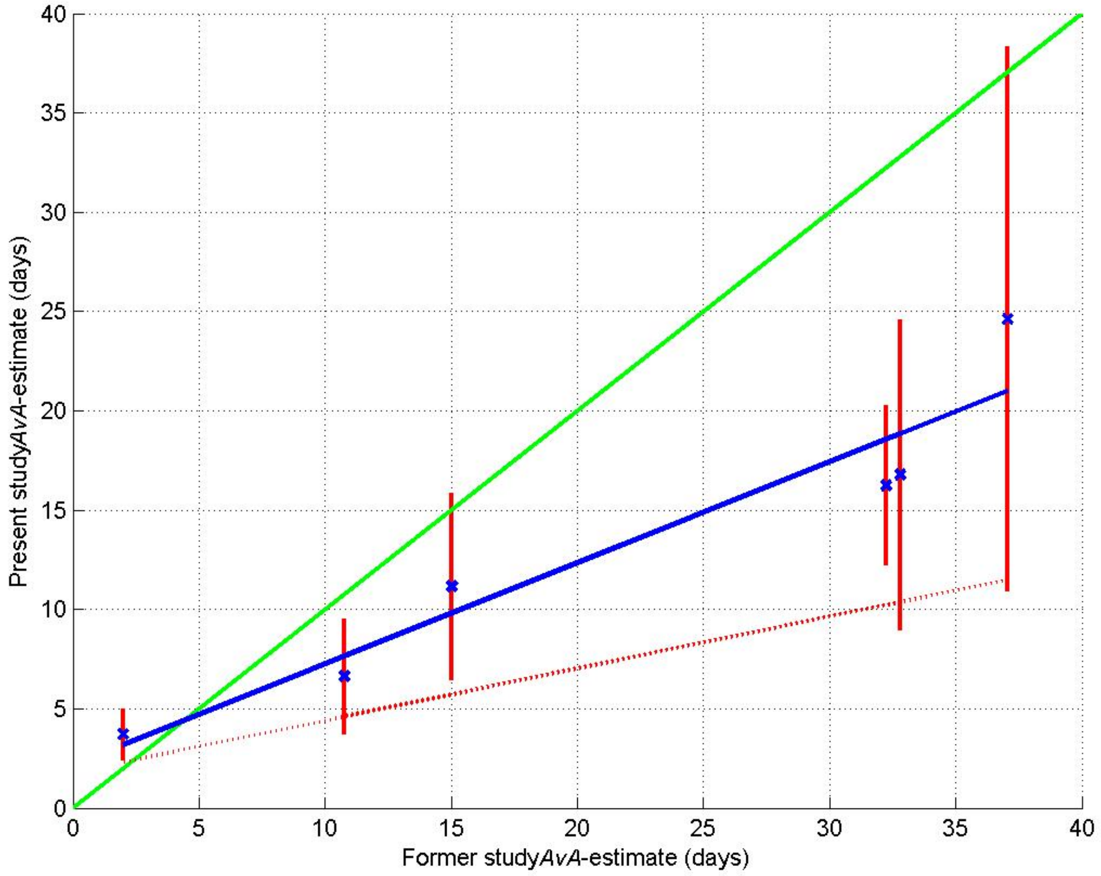

Fig. S7 Scatter diagram showing the AvA values of the present and former configuration SBs. The present study's strait hypsographic data are entirely based on DEM in combination with computed sedimentation processes, the former data are to a certain extent also based on soundings. The blue solid regression line involves all SBs that are deemed as sufficiently similar with regard to their horizontal delimitation to be pairs of a valid comparison. The red bars denote the S.D. of the present data and the red dotted line is formed as the regression line of the same data set minus 1 S.D. The slope of both regression lines is smaller than the diagonal equivalence line in green and the latter amounts to about 1/3 for the latter line

Table S1 Progressive land-lift transformation of the biosphere objects (BOs) from being a part of the open sea to becoming part of the land ecosystems is graphically presented. The upper section (BO 100 through 151) pertains to the Forsmark area for which the BC years were treated by the AS3D-model and not MIKE-3. BOs 201 through 216 belong to Laxemar-Simpevarp with the coastal zone (CZ) explicitly listed for this latter group. For BOs 201, 202, 203, 209, 213, and 216 temporary lakes are occurring. For 205, 209, and 216 these temporary lakes become coastal SBs again. Only the years 6500 BC and 2000 BC were not treated coincidentally and the corresponding columns are consequently left blank

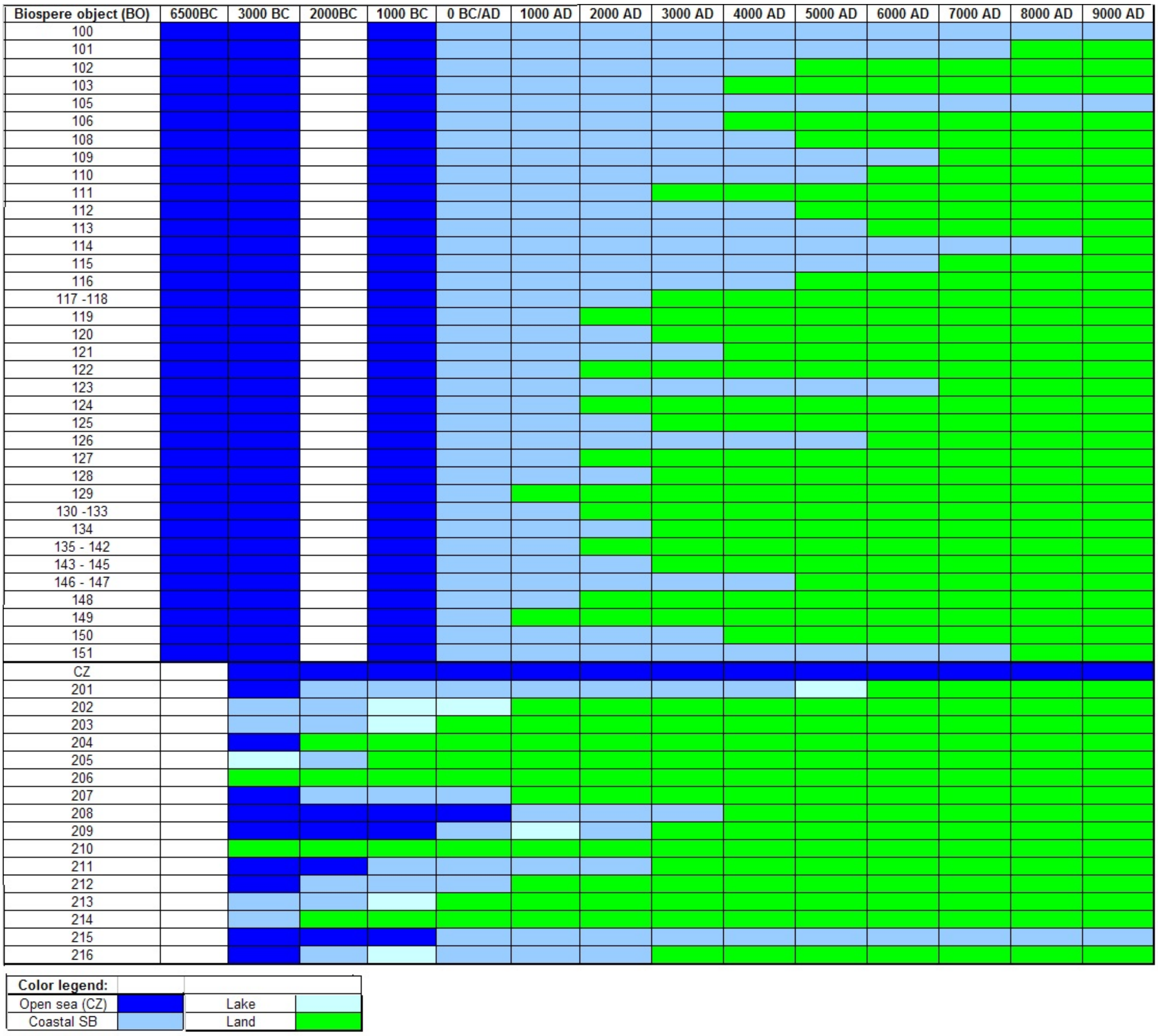

BOX S1: PRESENTATION OF THE MIKE3FM MODEL

The MIKE 3 system consists of several modules. The basic module, HD, simulates the hydrodynamic processes, describing the water movements based on the driving forces.

The HD module is based on the numerical solution of the three-dimensional, incompressible, Reynolds-averaged Navier-Stokes equations invoking the assumptions of Boussinesq and of hydrostatic pressure.

Thus, the model consists of equations for the continuity of mass, momentum, temperature, salinity and an equation of state. The set of equations is closed by a turbulent closure scheme.

The free surface is taken into account using a sigma-coordinate transformation approach (DHI 2011a, b). MIKE 3 FM accounts for all the important hydrodynamic processes, i.e.:

- Density stratification due to temperature and salinity variations
- Transport of salt and heat
- Density driven currents
- Bottom friction
- Wind forcing on the surface
- Currents driven by sea-level variations
- Freshwater runoff and cooling water discharge
- Heat exchange with the atmosphere
- Turbulence
- Coriolis force

The water volume in the area of interest is divided into a number of computational cells (a mesh), using a cell-centred finite volume method, in which sea level, currents, salinity, temperature, density and turbulence are calculated. In the horizontal plane, an unstructured mesh is used, whereas in the vertical the mesh is structured (see Figure S8, in this Box).

The mesh elements (cells) can be prisms whose horizontal faces are triangles. The mesh is tailored to accurately describe the bathymetry and shoreline.

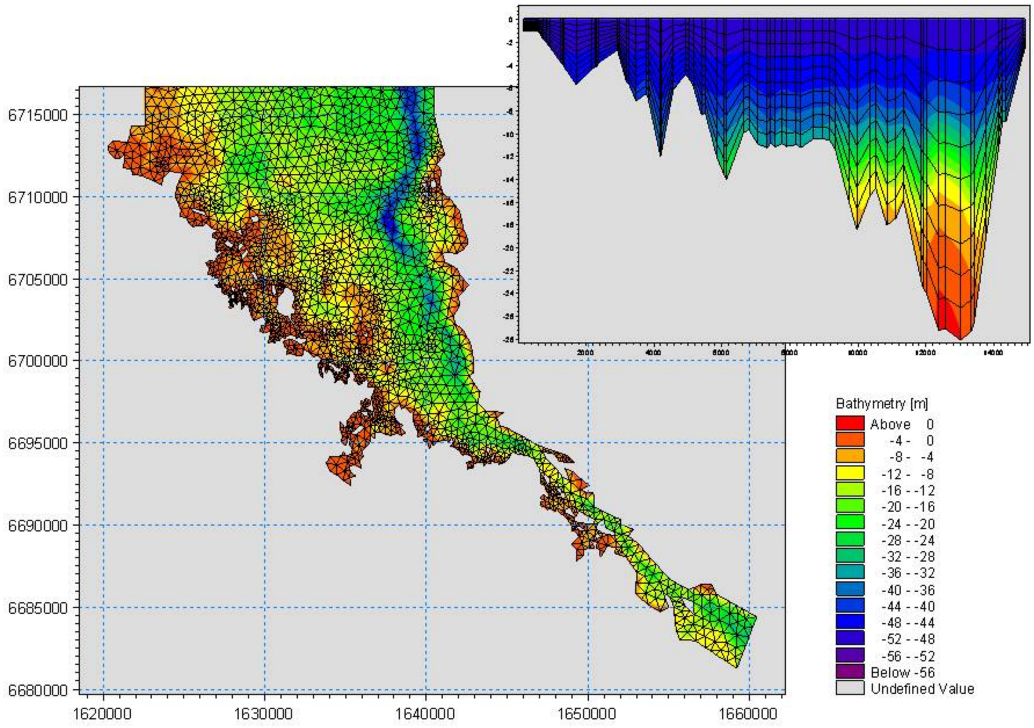

Fig. S8 Resolution of Öregrundsgrepen in the Forsmark area into irregular triangular grid elements. A cross-section transect across the bay in east-west direction is also shown, displaying the varying vertical positions of the σ-coordinates

BOX S2: OPEN COASTAL ZONE APPRECIATION OF AvA FOR FORSMARK CONCERNING BC TIMES

Three distinct phases in the prehistoric development of the Baltic Sea have been simulated for a full year cycle namely 6500 BC, 3000 BC and 1000 BC. The best-known data about the Baltic at those times is presumably the bathymetry. Forcing factors have mainly been the same as for a particular contemporary year 2004 with two exceptions: (i) the initial salinity distribution close to the surface layer of the Forsmark coast, for which prescribed values (Gustafsson and Westman 2002 and Gustafsson 2004) have been adhered to and (ii) the freshwater discharge.

Literature values have been used for the all-Baltic yearly discharge consistent with the estimated salinities. All other forcing data, in particular wind, pertain to the year 2004. The resulting 2D-velocity fields for the layer closest to the bottom at model grid points corresponding to the location of the Forsmark coast at the three above-mentioned periods together with appropriate length-scales yield the turnover times, which in turn can be converted to AvA estimates.

The representative mean velocities are found to be in the range of 2-3 cm/s. This means that the average residence time of hypothetically released nuclides would be determined by the time over which they are advected off the greatest sub-basin area (with a length-scale of three nautical miles) into which the corresponding location of the present Öregrundsgrepen is partitioned. This time-scale is found to be a little less than two days, i.e. somewhat longer than has been estimated for the present-day situation.

Box S3: THE CouBa MODEL HANDLING OF STRAIT EXCHANGE FLOWS AND CONSEQUENTIAL BASIN RESPONSES

The CouBa model computes the exchange flows through a strait given the stable density stratification in the basins on either side of the strait and the sea-level difference. The basins are considered to be horizontally homogeneous i.e., only resolved in the vertical direction.

Normally the sea-level difference is much smaller than the thickness of the stratified layers, but is nevertheless responsible for setting the less dense top layers in motion. The water layers in the basin with denser water flow in the opposite direction driven gravitationally by their comparatively greater density. In doing so they may drag even denser bottom water from beneath the height of the sill to pass through the strait by the so-called aspiration process.

At certain positive or negative sea-level differences the two-way exchange flow is replaced by a one-way exchange with possible stagnant layers still present at the strait constriction. The accordingly taxed and receiving basins respond to the exchanged fluxes and their inherent scalar properties (salinity, temperature and AvA) in addition to the induced vertical mixing, foremost caused by the local wind.

The heating and cooling processes through the surface are also an integral part of the model approach. The entailed basin responses (e.g. Stigebrandt, 2012) result in an altered stratification on either side of the strait when the next integration time step of the model is reiterated.

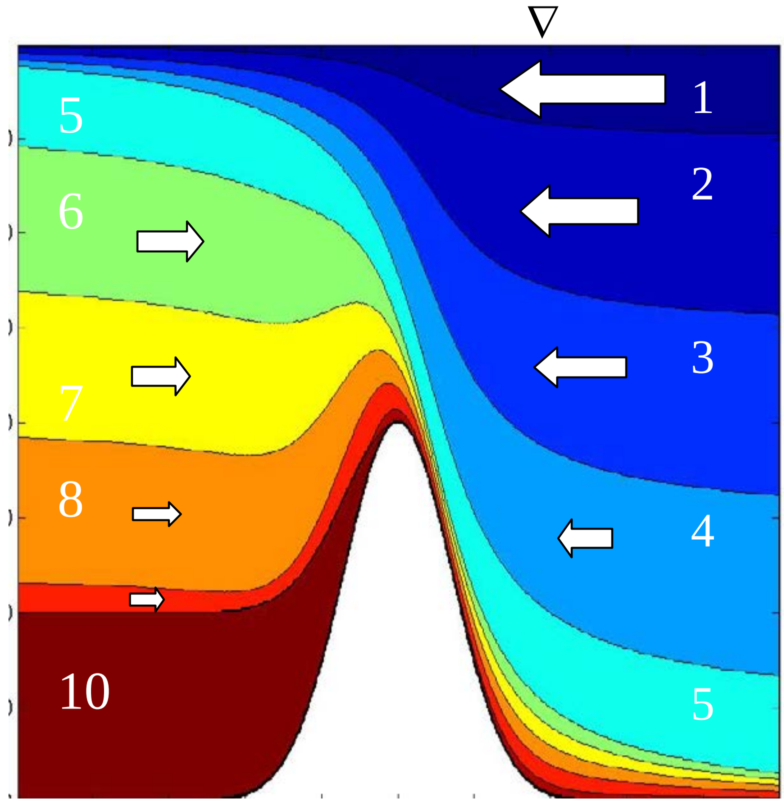

Fig. S9 Illustration of a multi-layer two-way exchange process with stably stratified layers on both sides of the strait. The less dense water is on the right side depicted in deeper blue colors and flows to the left due to a barely noticeable increased sea level on the right side. The arrows indicate the flow intensity and direction. For various sea-level differences one layer at a time becomes stagnant i.e. not flowing at all, which instances facilitate the computation of the layer volume flows w.r.t. the sea level difference. The light blue layer (#5) is in this instantiation assumed to be stagnant as is layer #10 which is barely aspired to pass through the strait

BOX S4: GUIDELINES FOR CONVERTING GIS DATA TO BASIN CONFIGURATIONS

1. If the locations of water areas one meter above mean water level (MW) are in the form of a simple conjoined area (see e.g. BO 214 on the map for 3000 BC, Figure 3b), such areas are denoted with the corresponding BO number ranging from 201 through 216. For the model computations, the SBs and straits in the configuration diagrams are relabeled in consecutive order with a leading letter B and S respectively.
2. If the appropriate partitioning of two adjacent basins, e.g. 205 and 207, from a coastal oceanographic point of view coincides with the delimitation between these basins, this strait is denoted 205/207 and is drawn in the configuration diagrams as black solid lines. Such borders coincide with thresholds.
3. If the water areas within a basin occur in several non-connected coastal basins or if there are coastal oceanographic motives to make a finer subdivision of a conjoined area, then these SBs are marked with an order number trailing the BO number after a hyphen sign. Such interfaces are marked both in maps and configuration diagrams (Figure 4 a-d) with dashed bold lines.
4. The peripheral SBs that are specifically to be pointed out as belonging to the coastal zone (CZ) are indicated with a trailing zero. A SB of the numbered basin that due to a sufficiently large section area of the strait interface is considered as belonging to the coastal zone is consequently denoted with a trailing zero after the basin number, e.g. 205-0.
5. Complicated and wide interfaces between two basins (e.g. 202 and 203 for 3000 BC, Figure 3b) may be a motive to conjoin them.
6. For physical oceanographic reasons it is strongly motivated to include auxiliary SBs that are outside the BO areas. Their inclusion acts to increase the residence times relative the CZ. Such new coastal SBs are denoted ’New1’, ‘New2’ (Figure 4) and so on until all needed auxiliary SBs are accounted for. More technical detail can be found in Engqvist (2010).

Box S5: **PROCEDURE FOR CONVERTING GIS DATA TO STRAIT CROSS-SECTION HYPSO-GRAPHIES**

For the ensuing straits interconnecting the SBs, the procedure is that the depth along the interface of two adjacent basins is extracted from the DEM. For straits with a decided main flow direction, their cross-section areas along the deduced delimiting sill ridges are projected perpendicularly to this main direction.

For more complicated arrangements with straits pointing in various directions and with varying widths, these have been treated individually and are accounted for as operating in parallel. The extraction of the strait hypsographic data starts with the strait interface plotted in the DEM coordinate system.

First, the coordinate system rotated so that the main flow direction is aligned with an x-axis.

Second, the cross-sections are projected on a y-axis. The total width for each meter downward (starting from 1m above MW level) is assessed. For the simple straits this data extraction can readily be done by algorithmic coding.

For more complicated straits graphical methods tend to be more efficient. More technical detail can be found in Engqvist (2010).

REFERENCES

DHI. 2011a. MIKE 21 & MIKE 3 flow model FM, hydrodynamic module: Short description. DHI, Hørsholm, Denmark, 12 pp. Retrieved March 9, 2013, from http://www.dhisoftware.com/Download/DocumentsAndTools/~/media/Microsite_MIKEbyDHI/Publications/PDF/Short%20descriptions/MIKE213_FM_HD_Short_Description.ashx

DHI. 2011b. MIKE 21/3 Ecological modeling: MIKE 21/3 ECO Lab FM Module: Short description. DHI, Hørsholm, Denmark, 14 pp. Retrieved March 9, 2013, from http://www.dhisoftware.com/~/media/Microsite_MIKEbyDHI/Publications/PDF/Short%20descriptions/MIKE213_FM_EL_Short_Description.ashx

Engqvist, A. 2010. Estimation of residence times of coastal basins in the Laxemar-Simpevarp area between 3000 BC and 9000 AD. Svensk Kärnbränslehantering AB, SKB R-10-57, Stockholm, Sweden, Report, 64 pp.

Gustafsson, B. 2004. Millenial changes of the Baltic Sea salinity. Studies of the sensitivity of the salinity to climate change. Svensk Kärnbränslehantering AB, SKB TR-04-12, Stockholm, Sweden, Report, 42 pp.

Gustafsson, B., and P. Westman. 2002. On the causes for salinity variations in the Baltic during the last 8500 years. *Paleooceanography* 15**:** 1-12.

Stigebrandt A. 2012. Hydrodynamics and circulation of fjords. In *Encyclopedia of lakes and reservoirs*, eds. L. Bengtsson, R.W. Hershy, and R.W. Fairbridge, pp. 327-344. Berlin: Springer Science+Business Media B.V. doi: 10.1007/978-1-4020-4410-6
